# IVUS Longitudinal and Axial Registration for Atherosclerosis Progression Evaluation

**DOI:** 10.3390/diagnostics11081513

**Published:** 2021-08-22

**Authors:** Nikos Tsiknakis, Constantinos Spanakis, Panagiota Tsompou, Georgia Karanasiou, Gianna Karanasiou, Antonis Sakellarios, George Rigas, Savvas Kyriakidis, Michael Papafaklis, Sotirios Nikopoulos, Frank Gijsen, Lampros Michalis, Dimitrios I. Fotiadis, Kostas Marias

**Affiliations:** 1 Computational BioMedicine Laboratory, Institute of Computer Science, Foundation for Research and Technology Hellas—FORTH, 70013 Heraklion, Greece; kspan@ics.forth.gr; 2 Department of Biomedical Research, Institute of Molecular Biology and Biotechnology—FORTH, University Campus of Ioannina, 45115 Ioannina, Greece; panagiota.tsompou@gmail.com (P.T.); g.karanasiou@gmail.com (G.K.); giannakara55@gmail.com (G.K.); ansakel13@gmail.com (A.S.); savvasik21@gmail.com (S.K.); fotiadis@uoi.gr (D.I.F.); 3 Unit of Medical Technology and Intelligent Information Systems, Department of Materials Science and Engineering, University of Ioannina, 45110 Ioannina, Greece; george.a.rigas@gmail.com; 4 Department of Cardiology, Medical School, University of Ioannina, 45110 Ioannina, Greece; m.papafaklis@yahoo.com (M.P.); soter1313@yahoo.gr (S.N.); lamprosmihalis@gmail.com (L.M.); 5 Department of Cardiology, Erasmus University Rotterdam, 3062 PA Rotterdam, The Netherlands; f.gijsen@erasmusmc.nl; 6 Department of Electrical and Computer Engineering, Hellenic Mediterranean University, 71004 Heraklion, Greece

**Keywords:** atherosclerosis, IVUS, stent, longitudinal registration, axial registration, image registration, 3D registration, ultrasound

## Abstract

Intravascular ultrasound (IVUS) imaging offers accurate cross-sectional vessel information. To this end, registering temporal IVUS pullbacks acquired at two time points can assist the clinicians to accurately assess pathophysiological changes in the vessels, disease progression and the effect of the treatment intervention. In this paper, we present a novel two-stage registration framework for aligning pairs of longitudinal and axial IVUS pullbacks. Initially, we use a Dynamic Time Warping (DTW)-based algorithm to align the pullbacks in a temporal fashion. Subsequently, an intensity-based registration method, that utilizes a variant of the Harmony Search optimizer to register each matched pair of the pullbacks by maximizing their Mutual Information, is applied. The presented method is fully automated and only required two single global image-based measurements, unlike other methods that require extraction of morphology-based features. The data used includes 42 synthetically generated pullback pairs, achieving an alignment error of 0.1853 frames per pullback, a rotation error 0.93° and a translation error of 0.0161 mm. In addition, it was also tested on 11 baseline and follow-up, and 10 baseline and post-stent deployment real IVUS pullback pairs from two clinical centres, achieving an alignment error of 4.3±3.9 for the longitudinal registration, and a distance and a rotational error of 0.56±0.323 mm and 12.4°±10.5°, respectively, for the axial registration. Although the performance of the proposed method does not match that of the state-of-the-art, our method relies on computationally lighter steps for its computations, which is crucial in real-time applications. On the other hand, the proposed method performs even or better that the state-of-the-art when considering the axial registration. The results indicate that the proposed method can support clinical decision making and diagnosis based on sequential imaging examinations.

## 1. Introduction

Intravascular Ultrasound (IVUS) is an imaging technique for visualizing the internal vascular structure in order to assist with the diagnosis of the atherosclerotic disease giving information about the stenosis, as well as the plaque composition in the arterial wall. IVUS pullbacks are obtained using an ultrasound transducer attached to a catheter which is mechanically dragged inside the arterial vessel (from the distal to the proximal position). IVUS can be used in a temporal fashion to assess physiological changes. In addition, IVUS 2D cross-sectional vessel images can also be used to reconstruct a 3D representation of the vessel. Comparison of IVUS pullbacks acquired at two time points enable the estimation of the disease progression over time. In order for this to be achieved, it is often the case that two IVUS pullbacks need to be compared by the healthcare professional, which is a time consuming process.

**Figure 1:**
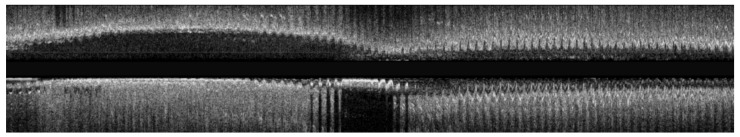
Sagittal views of an IVUS pullback prior to the sub-sampling of the frames corresponding to the end-diastolic cardiac phase.

On the other hand, temporal IVUS image pullbacks suffer from artifacts and image variations that are caused by cardiac pulsation, which can complicate their analysis rendering the comparison of temporal datasets hard. First, the transducer is affected by an oscillation in the longitudinal axis, due to the periodic cardiac muscle contraction and expansion. Such oscillations result in some vessel’s cross-sections to be sampled multiple times during one pullback, which can be observed as sawtooth-like artifacts in the longitudinal reconstruction of the pullback [1], as seen in Figure 1. In addition, heart contraction causes a non-rigid deformation of the vessel due to its compliance. As a result the appearance of a given IVUS cross-section depends on the sampling instant with respect to the heart cycle. Therefore, to compare the same cross-section of the vessel over time, it is essential to select the corresponding frames with regard to the cardiac cycle. Moreover, due to the catheter being withdrawn through the blood vessel, its axial position with respect to the vessel is not fixed and the catheter freely moves inside it leading to rotational inconsistencies between temporal acquisitions. As a consequence, some vessel sections may be captured multiple times while several frames might be misaligned both longitudinally and axially.

To enable the efficient comparison of temporal IVUS pull-back sequences, longitudinal and axial registration is required to allow the clinicians to effectively assess any physiological changes. Currently, there are limited studies focusing on IVUS longitudinal registration while small datasets are used. In addition, most registration methods related to IVUS pullbacks usually deal with either longitudinal [2,3] or axial registration [4,5] exclusively. Longitudinal registration is commonly accomplished using the Dynamic Time Warping (DTW) algorithm [2,3]. In [6], temporal and axial registrations are combined into a single pipeline using a 3D DTW algorithm, which focuses on finding not only the corresponding images between the IVUS pullbacks, but also the rotation angle that aligns the matched images. All methods that utilize the DTW algorithm use a cost function to calculate the similarity between two frames. Such cost function may be based on features derived from vessel or lumen areas [7] or vessel’s bifurcations [8]. The extraction of these features is based on segmenting and analyzing the morphological properties of such areas [9]. In addition, instead of using only shape related features or intensity-based methods [4], a combination of them can be used [6] for longitudinal or axial registration. Apart from the DTW, several researchers utilize other mathematically-oriented methods, such as splines [8] or likelihood estimation [10] of possible correspondences between the two pullbacks for longitudinal registration. In the axial registration, the comparison is estimated via features [7], pixel intensity [4] or both [8]. In addition, a recent study proposed an unsupervised method based on deep learning to axially register pairs of IVUS pullbacks [5].

In this study, we propose a method for the longitudinal and axial registration of pairs of IVUS pullbacks, with emphasis in prior and post stent IVUS matching. The first step regards the longitudinal registration and is based on a variation of the DTW algorithm, which matches each pair of corresponding frames in the pullbacks with one another. The second step regards the 2D axial image registration of each matched pair of images, and is formulated as an optimization problem. Although the longitudinal registration performs worse than the state-of-the-art (see Table 1 for a quantitative comparison), it still achieves a mean alignment error of 4.3±3.9 frames per pullback which is comparable to [7]. On the other hand, the axial registration performs outperforms the state-of-the-art (see Table 2 for a quantitative comparison), with a distance error of 0.56±0.323 mm and a rotational error of 12.4°±10.5°. The results show that the longitudinal and axial registration can be achieved up to a significant extent without the need for laborious and time-consuming preprocessing steps or morphology-based feature extraction, utilizing a simple similarity function at the pixel level. The novelty of this study is that unlike other methods that require computationally heavy preprocessing and morphology-based feature extraction, our method is lightweight, which is a requirement for online IVUS-IVUS pullback examination, relying on a simple noise filtering and two global image-based features (i.e., cross correlation measurement for the longitudinal registration and mutual information measurement for the axial registration). In addition, we propose a fully automated method, which incorporates the registration on both the longitudinal and axial axis.

## 2. Problem Formulation

### 2.1. Data Preparation

As discussed in the introduction, the contraction and expansion of the heart muscles lead to periodical deformations of the vessel structures as well as to longitudinal oscillation of the IVUS catheter. As a result, the captured imaging sequences suffer from time varying deformations and repetitive frames across the entire section of the vessel, which depend on the cardiac cycle (Figure 1). In order to reduce these effects and address the heart cycle dependent vessel appearance, a downsampling method on the IVUS pullback is applied through a gating method. The rationale is that by sampling frames at a given point of the cardiac cycle, the resulting pullbacks convey more consistent vessel morphology information [1]. Various gating methods based on electrocardiogram (ECG) signals have been developed and effectively applied to such pullbacks. By analyzing the ECG signal either during the procedure (online) or afterwards (offline), the heart cycle can be accurately determined and frames that correspond to a particular point of the cardiac cycle can be selected [11]. When the ECG signals cannot be obtained or when they are noisy and thus do not perform well, a ECG-like signal can derive by analyzing the IVUS pullbacks themselves [12]. Finally, when none of the above signals are present, the sub-sampling can be performed manually by an expert physician. In this study, for the pullbacks, the reference time point of the heart cycle was the end-diastolic phase, during which the heart is almost stationary, while those images that did not correspond to the end-diastolic phase of the cardiac cycle were not included in the dataset. As a result, the coronary arteries, which are located on the surface of the heart, are relatively immobile at the end-diastolic phase. Having achieved this important data preparation step, the proposed algorithm is designed to address the remaining problems regarding multiple frames of the same anatomical area and rotational inconsistencies.

### 2.2. IVUS Pullbacks Registration

**Figure 2:**
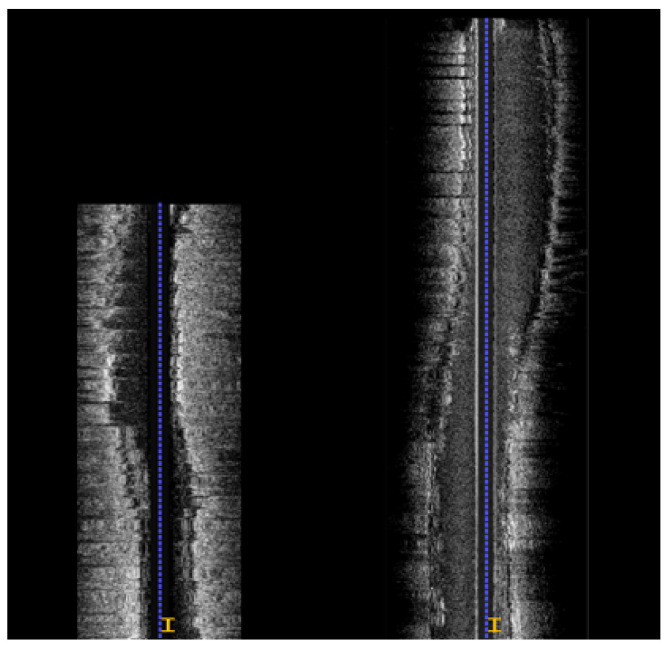
Pre stent IVUS series (left) and post stent IVUS series (right). It is obvious that the two pullbacks differ in length, due to them either having different starting and ending points or some regions of the vessel having been captured multiple times.

In order to compensate for the remaining incoherencies, two registration steps are followed (longitudinal and axial registration), after the selection of the end-diastolic frames of each sequence. Although downsampling the sequences and the selection of the end-diastolic frames partially addresses the issue of the oscillations of the catheter, a longitudinal alignment of the captured IVUS sequences of a particular vessel is still mandatory. There are two main reasons regarding the need for longitudinal registration: (i) the beginning and ending frames of two corresponding IVUS sequences may represent different regions within the vessel. Thus, one needs to align the sequences so that such starting and ending points of one sequence match with the corresponding points of the other one (Figure 2). This may lead to partial overlapping, (ii) due to the periodical oscillations of the catheter, a particular cross-section may be captured more than once. Thus, a one-to-one image correspondence between the two sequences may not be always possible and an image frame from one sequence may correspond to multiple frames in another sequence.

Following the longitudinal registration step, a 2D axial registration between the matched frames of the sequences needs to be performed, due to the misalignment of the corresponding frames of the two pullbacks in the 2D plane caused by rotational displacements of the catheter while moving freely within the vessel during pullback, as previously discussed. Notably, since non-rigid, time-varying deformations have been eliminated by the downsampling method of selecting only the images corresponding to the end-diastolic phase of the cardiac cycle, we focus on rigid registration techniques instead of more complex ones, such as affine and non-rigid ones.

## 3. Proposed Registration Pipeline

**Figure 3:**
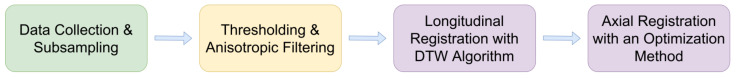
IVUS pullbacks registration pipeline.

In this section we present the pipeline of the proposed methodology (Figure 3), which consists of three stages:

1. Data Preprocessing: Image processing methods are employed for the removal of unnecessary artifacts and noise.
2. Longitudinal Registration: The corresponding image pairs of the two IVUS image sequences are selected.
3. Axial Registration: Axial alignment of the corresponding images using rigid image registration is performed.

### 3.1. Data Preprocessing

The proposed pipeline includes a preprocessing stage, prior to the registration stage. In particular, two filtering operations are applied on each image of each pullback. First, a thresholding operation is applied to eliminate the low-intensity artifacts, such as those due to the illumination of the blood or the catheter’s probe. The threshold value was set to the 90th percentile of the Cumulative Distribution Function (CDF) histogram leading consistently to improved image appearance. In addition, an anisotropic diffusion filter was applied [13,14], to reduce the speckle noise without blurring important image information such as its edges, as the Gaussian filtering would do [15]. This way, IVUS images become less noisy and more homogeneous which is important for improving the performance of the automated registration method proposed.

### 3.2. Longitudinal Registration

For the longitudinal registration, a DTW based algorithm is applied. Although DTW was first introduced as a method for time series alignment [16], it can be used on longitudinal imaging sequences, with the proper adaptation of the cost function. For the formulation of a mathematical cost function that can be used with imaging data, we utilize the Cross Correlation (CC) to measure the similarity between the two images in question. The cost function is calculated as in (1) and its values are in the [0,1] range.

(1) d(i,j)=1−|CC(i,j)|,

where *i*, *j* are the i-th and the j-th frames of the first and second IVUS pullbacks respectively. The reason for using this dissimilarity metric is to remove the need for further image processing such as segmentation of the lumen or the atherosclerotic plaque, which can be computationally costly and time-consuming. Finally, the DTW matrix, *D*, is computed:

(2) $$\begin{array}{cc} D(i,j)=d(i,j)+min(D(i,j-1) & +C,D(i-1,j)+C,\\ \; & D(i-1,j-1)), \end{array}$$

where *C* is an added regularization cost, which is used in order to favor the diagonal options of the warping path instead of the vertical and horizontal ones. Doing so, less frames of one sequence match with multiple frames of the other and the smoothness of the output warping path increases. The full description of the algorithm is beyond the scope of this paper and its detailed description can be found in [3].

### 3.3. Axial Registration

Given the longitudinally registered sequences, each matched pair of images must be registered with respect to one another in the spatial 2D pixel domain. We follow an intensity-based registration method, which is formulated as an optimization problem whose purpose is to estimate the optimal rigid transformation needed to align each pair of images. In particular, our method estimates the transformation that maximizes the Mutual Information between a pair of images by using a variant of Harmony Search (HS) as an optimizer [17].

#### 3.3.1. Mutual Information

Mutual Information (MI) [18] has been used extensively in image registration as a similarity metric. Unlike other metrics, e.g., Cross Correlation, MI does not assume a linear relationship between the two sets of data and is described as in Equation (3):

(3) $$MI\left(T(F),R\right)=\sum_{f\in F,r\in R}p_{RF}\left(f,r\right)\log\frac{p_{RF}\left(f,r\right)}{p_{R}\left(r\right)\;\cdot \;p_{F}\left(f\right)},$$

where *F*,*R* are the floating and reference image respectively, *f*, *r* are the pixel intensities of the floating and reference image respectively and T describes the transformation of the floating image. The mathematical function of MI is highly non-convex, which makes it difficult to find the optimum transformation, i.e., the transformation that aligns the images. The optimization method must be simple and efficient and therefore, the Harmony Search optimizer was used.

#### 3.3.2. Harmony Search Optimizer

Harmony Search is a relatively simple meta-heuristic optimization method. It constructs each new solution (i.e., transformation matrix) by utilizing elements either from existing solutions in the current candidate population (i.e., its memory) or by using random elements sampled from the search space, Equation (4).

(4) $$new\_sol\left[i\right]=\left\{\begin{array}{c} k\left[i\right],\;with\;probability\;h\left(1-p\right)\\ k\left[i\right]+e,\;with\;probability\;hp\\ U\left(Min_{i},\;Max_{i}\right)\;with\;probability\;\left(1-h\right) \end{array}\right.,$$

where *i* is the i-th element of the solution, *k* is a randomly chosen solution from the current population, *e* is a random step for slight tuning, h is the probability to use a solution from the current population, *p* is the probability to slightly change an element from a chosen solution, $U\left(Min_{i},\;Max_{i}\right)$ is a uniform distribution whose upper limit is the Maximum value of the i-th element and the lower limit is the minimum value of the i-th element.

By constructing a new solution based on multiple previous ones, Harmony Search can produce more possible solutions than other optimization methods can. In addition, due to the use of the Uniform Distribution, Harmony Search avoids converging to local minima while it quickly converges to the global minimum [17].

In this work, a variant of Harmony Search is used [17] for faster convergence. The random step *e* is replaced with a step that is calculated using aspects of the A.L.O.P.E.X algorithm [19,20] which combines the advantages of Simulated Annealing and Gradient Descent. Additionally, the values of *h* and *p* gradually increase until a certain maximum value, which facilitates exploration of the search space rather than exploitation, for faster convergence.

## 4. Results

**Figure 4:**
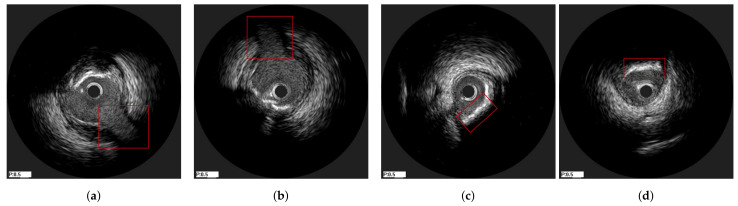
Two corresponding pairs of annotated frames based on the presence of a bifurcation (**a**,**b**) and calcification (**c**,**d**), which are highlighted with a red box in the images.

The method was tested on IVUS data from 21 patients from two clinical centres, i.e., University Hospital of Ioannina, Greece and Erasmus EMC, Netherlands. The data of 10 patients consisted of two IVUS pullbacks, corresponding to a pre- and a post-stent deployment (immediately after the procedure) pullbacks respectively. For the remaining 11 patients, the first pullback was from a pre-stent deployment examination and the second one from a follow-up IVUS examination (after a few months). As a result the dataset used was heterogeneous which was a prerequisite for testing the robustness of the proposed method. The mean pullback length was 118 frames. In this study, two different experiments were conducted. The first experiment regarded the evaluation of the method on synthetic data, for which the ground truth information for each registration stage was available. The second experiment was based on the in-vivo data, for which ground-truth frame correspondence was available for the longitudinal registration based on the presence of specific landmarks (i.e., bifurcations and calcifications), which were easy to be detected across IVUS examinations. These landmarks were annotated by an expert cardiologist based on careful examination of each pullback pair. In addition, the location of each landmark was marked using a manual annotation tool by the expert cardiologist to be used as ground truth when evaluating the axial registration. Figure 4 presents an example of corresponding frames with two annodated landmarks (a bifurcation and a calcification).

### 4.1. Experiments on Synthetic Data

Pairs of sequences were synthetically generated by modifying a single pullback in order to create IVUS pairs. Since there were two pullbacks for each of the 21 patients, 42 synthetic pullback pairs were generated. To generate the synthetic images we applied combinations of the following distortions:

1. Amplitude distortion, by adding random zero-mean Gaussian noise to the image.
2. Partial overlapping, by discarding a subset of the pullback at its beginning and its ending. The length of the overlap was between 60–80% of the original size, randomly sampled based on the uniform distribution.
3. Longitudinal distortion, by randomly repeating an image of the pullbacks to simulate the longitudinal oscillation of the probe. Each image in the pullback had a 10% probability to be repeated, and the times of that repetition was randomly sampled between 1–4 based on the uniform distribution.
4. Rigid distortion, by randomly translating and rotating an image to simulate the circumferential movement of the probe. The rotation range was [−15°,15°], the translation range is [−5,5] pixels and both are sampled based on the uniform distribution.

**Figure 5:**
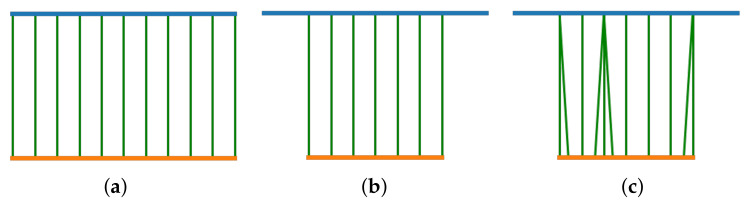
Example of the synthetic data. Blue line is the reference pullback, orange line is the synthetic pullback and green lines indicate the correspondences. (**a**) Original pullbacks with original 1-1 correspondences, (**b**) after applying overlapping distortion, (**c**) after repetition of some frames.

An intuitive example of the longitudinal distortions (i.e., steps 2 and 3) is illustrated in Figure 5.

To evaluate the performance of the method the alignment error regarding the longitudinal registration was computed, i.e., the average distance between the reference and the computed warping path. In addition, we computed the error of the rigid transformation (rotation and translation) between each pair of corresponding images, to evaluate the axial registration on the synthetic dataset.

The results for the longitudinal registration are presented in Table 3, alongside with the performance results of other methods applied on synthetic datasets reported in the literature. In fact, our method outperformed other studies, with an alignment error 0.0942 on a similarly developed synthetic dataset regarding the longitudinal registration (i.e., only considering distortions 1–3), as seen in Table 3. However, it should be pointed out that such a direct comparison should be done with caution, due to heterogeneous datasets between the studies. Overall, the method presented in this paper achieved an average alignment error a 0.1853 frames per pullback for a mean pullback length of 118 frames, a rotational error 0.93°±2.3° per frame and a translational error in terms of Euclidian distance of 0.67±1.53 (in pixels) and 0.0161±0.0367 (in millimeters) per frame on the synthetic dataset with all distortions applied.

### 4.2. Experiments on In-Vivo Data

Regarding the evaluation of the longitudinal registration on the in-vivo data, Table 4 presents the alignment error in frames based on the landmark pairs annotated by the expert cardiologist. The proposed method was able to successfully detect and match most of the annotated frames with a mean alignment error 4.3±3.9 frames per pullback. The higher mean frame errors were observed on pullbacks where the number of frames of the registered pullback was also large. That is why Table 4 also includes a normalized error metric defined as the ratio of the mean error to the length of the registered pullback. In addition, Table 5 presents the distance and rotational error regarding the axial registration between the frames with the annotated landmarks. The axial registration achieved a mean distance error of 0.56 mm and a rotational error of 12.4°.

In addition, we computed the Mutual Information (MI) metric in three different settings:

1. Mean MI of the corresponding frames in the overlap of the unregistered end-diastolic IVUS pullbacks starting from the first frame of each sequence. (blue)
2. Mean MI of the matched frames of the longitudinally registered sequences. (red)
3. Mean MI of the 2D (axially) registered frame pairs of the longitudinally registered sequences. (grey)

**Figure 6:**
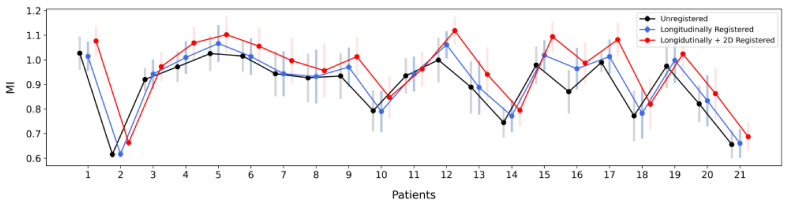
Boxplots of Mutual Information (MI) similarity metric across patients at all registration stages.

The results are presented in Figure 6. The increase of the mean image similarity between the corresponding frames of the pre- and post-stent IVUS pullbacks in all patients across the three experiment settings further indicated that each registration stage improved the alignment of the longitudinal image pairs. On average, the mean MI increased by 2.23% after the longitudinal registration, and by an additional 4.67% after the axial registration.

**Figure 7:**
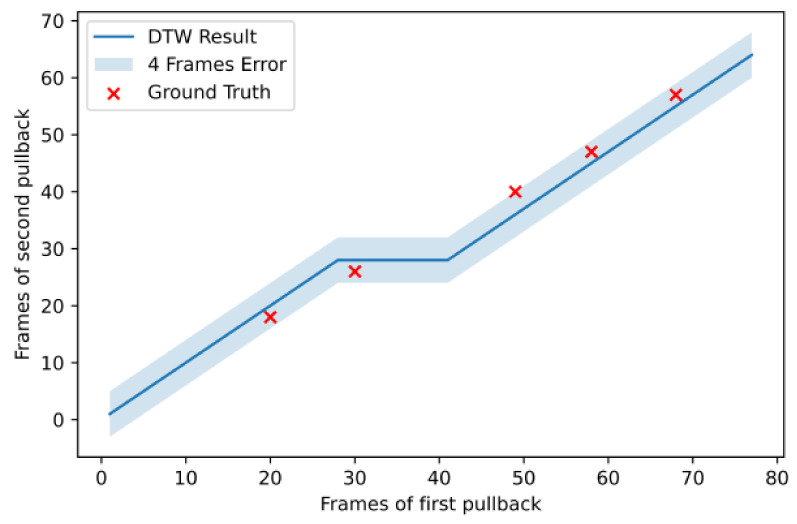
Longitudinal registration outcome for patient 6.

**Figure 8:**
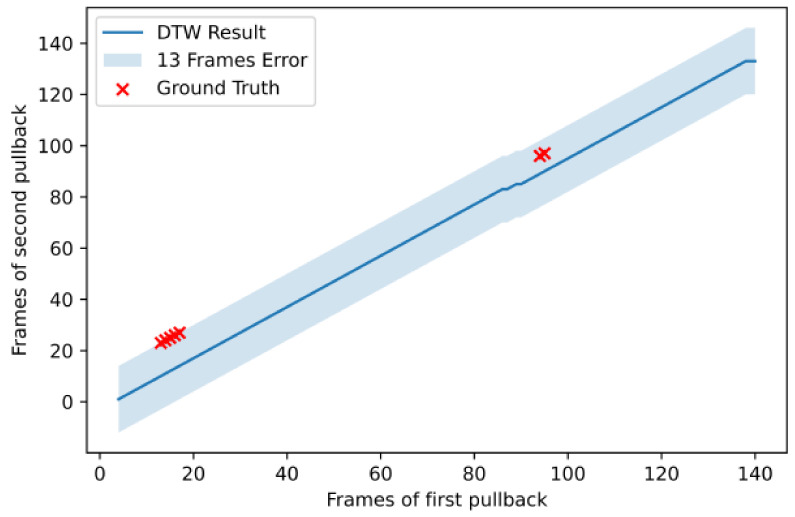
Longitudinal registration outcome for patient 18.

**Figure 9:**
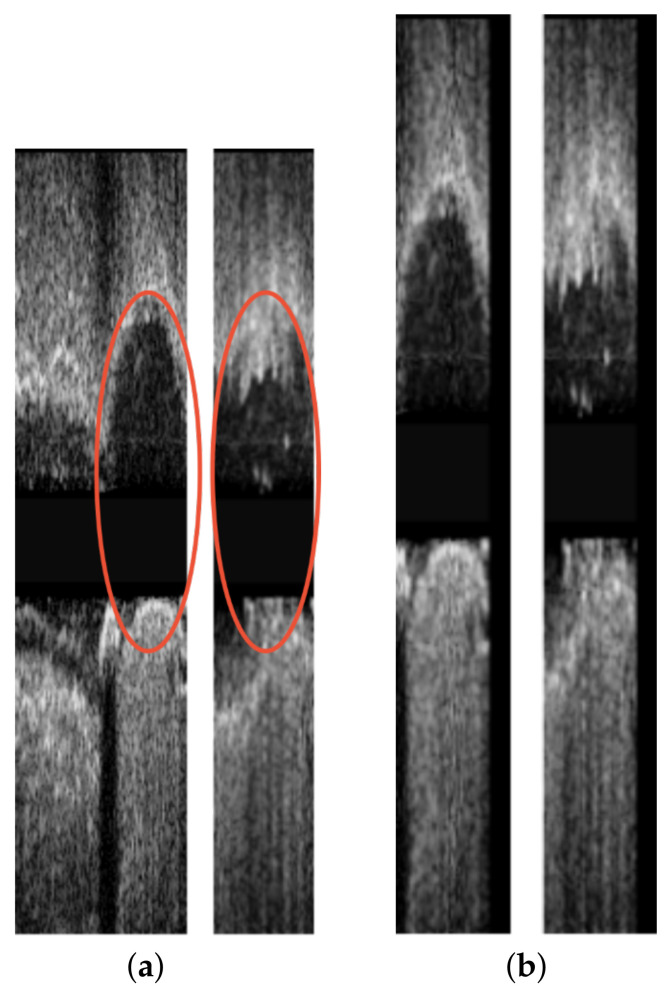
Sagittal vessel view corresponding to the (**a**) unregistered pullbacks (**b**) longitudinally registered pullbacks of patient 3. The left image of each subfigure corresponds to the pre-stent deployment pullback, and the right one to the post-stent deployment pullback. The red circles indicate the corresponding parts of the vesels.

An example from a longitudinal registration (DTW algorithm) from one case on which the method performed very well is presented in Figure 7, while Figure 8 present the worst case among the dataset. We can observe that, although the maximum error for the patient 18 was 13 frames, its total number of frames was also twice the number of frames of patient 6 (i.e., ≈140 vs. ≈70), which made the longitudinal registration step more difficult and prone to error. In addition, the sagittal vessel views across each registration step of another case are shown at Figure 9. By observing the sagittal view of the unregistered pullbacks (Figure 9a), it becomes evident that the longitudinal registration step (Figure 9b) matched the corresponding vessel regions highlighted (with an orange eclipse) in the unregistered pair in Figure 9a. Although longitudinal correspondence was established, there was still a need to account for rotational inconsistencies and this was achieved in the axial registration step.

**Figure 10:**
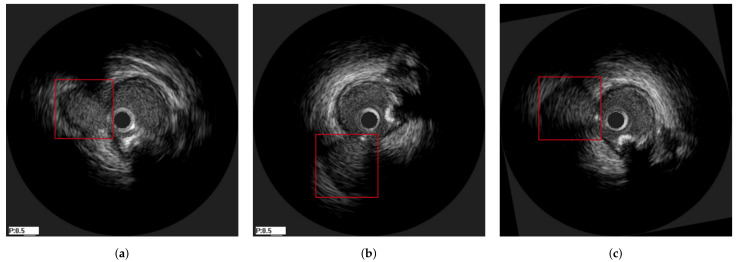
The alignment of a bifurcation can be observed under the marked area. (**a**) a frame from the first pullback of patient 6, (**b**) its corresponding frame from the second pullback of patient 6, (**c**) the axially (2D) registered frame from Figure 10b with respect to Figure 10a.

**Figure 11:**
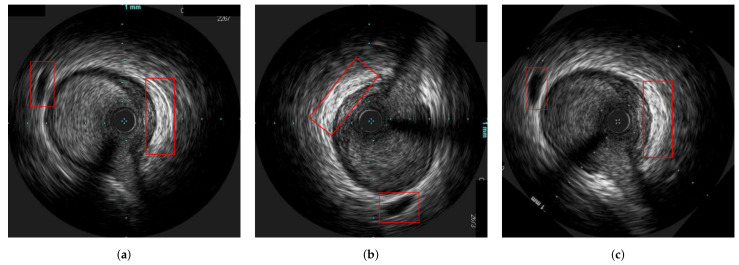
The alignment of the landmarks can be observed under the marked areas. (**a**) a frame from the first pullback of patient 18, (**b**) its corresponding frame from the second pullback of patient 18, (**c**) the axially (2D) registered frame from Figure 11b with respect to Figure 11a.

**Figure 12:**
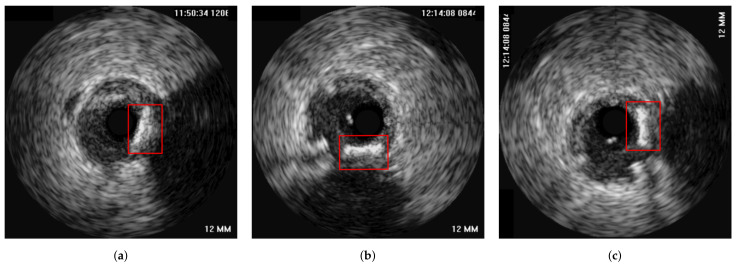
The alignment of the landmarks can be observed under the marked areas. (**a**) a frame from the first pullback of patient 3, (**b**) its corresponding frame from the second pullback of patient 3, (**c**) the axially (2D) registered frame from Figure 12b with respect to Figure 12a.

In Figure 10, an explanatory result of the axial registration step following longitudinal registration for a pair of IVUS images of the two pullbacks is presented. Figure 10a,b present longitudinally registered frames of the first and second pullbacks of patient 6 (patient 18), respectively, while Figure 10c is the result of the axial registration between (a) and (b). Two other similar examples are depicted in Figure 11 and Figure 12. The result showed that the images were further aligned and the anatomical correspondence of the vessels structures was improved. The match was not perfect due to the existence of non-linear changes in the vessels such as extension of the vessel after stent deployment or increase of the size of the plaque, which could not be fully aligned with rigid registration techniques. Another reason is that the end-diastolic matched pairs may not depict exactly the same area, but rather neighboring ones. The reason for using rigid registration was because we wanted to achieve an overall alignment of the frames without altering the actual shape of the vessel’s structures as a non-rigid deformation would do. To this end, the obtained results as highlighted in Figure 9, Figure 10, Figure 11 and Figure 12, demonstrated that the proposed registration framework was a fast and robust tool to establish a more intuitive comparison in temporal IVUS pairs.

## 5. Discussion

### Method, Novelty and Results

In this paper we propose a two-stage registration method, unlike some other studies [2,3,4], in order to both longitudinally and axially (2D) align a pair of IVUS pullbacks. First, the frames that correspond to the end-diastolic phase of the heart cycle were sampled by a physician due to the lack of syncrinous ECG aquisition. Then, we developed a DTW-based algorithm to longitudinally align the pullbacks. While comparison with previous methods is hard due to the lack of a common benchmark dataset, we performed a detailed review of previous similar methods and compared our results as much as it was possible.

Another novel part of this study regards the use of a highly heterogeneous dataset, consisting of pullbacks captured prior to and immediately after the stent deployment but also follow-up examinations after a few months of the procedure. Notably, even if the time interval of the pre-stent and follow-up IVUS examinations is quite long (e.g., 12 months which is typical in a clinical setting), our method performs comparably better to the pre- and post-stent examinations, having a mean alignment error 3.5 frames for the long interval examinations in contrast to the short intervals which have a mean alignment error 5.3 frames. That indicates that the proposed method is robust and that the structural changes in the vessels that occur between the pre-stent and follow-up examinations do not affect its performance.

Table 6 presents information related to the dataset’s size, the availability of ground truth and synthetic data while it is also compared to the datasets used in related studies. It is evident that the dataset used in this study is comparable to those used in other related studies, both in terms of its size as well as the availability of ground truth information, however an advantage compared to the state of the art is that the conducted experiments have been additionally applied on synthetic data.

Table 1 and Table 2 present a comparison between the performance of our method and that of other reported studies. Regarding the longitudinal registration, our method has a three times higher alignment error than the state-of-the-art which can be partly attributed to some cases being very hard to register (e.g., patients 15 and 18) as well as to the heterogeneity of the dataset. However, regarding the axial registration, our method outperforms the state-of-the-art regarding the mean distance error by 0.187 mm, while achieving comparable performance regarding the mean rotational error with a difference 3.33°. These comparative results should be however examined with care due to the lack of a common benchmark dataset which renders direct comparison hard. One unknown factor is the degree of data heterogeneity in other studies since the complexity of our dataset was very high containing pullbacks of patients after stent deployment as well as pullbacks captured after a long period of time in a follow-up examination where the morphology of the vessel’s structures may have changed significantly. Based on the above considerations, our method results in a satisfactory performance while it does not require computationally heavy preprocessing and morphology-based feature extraction prior to the registration step, as the other methods do. For this reason, the presented method can be a simple and fast diagnostic aid tool for cardiologists to better compare temporal IVUS sequences and assess the progression of the disease.

IVUS sequences are often compared in order to assess the intervention outcome in terms of e.g., lumen size, blood flow, stent positioning etc. While the necessity of a comparison tool to facilitate and enhance the comparison of temporal IVUS sequences is an unmet clinical need, only a limited number of studies have been presented to date. This is due to the fact that IVUS sequences suffer from inherent acquisition variability and noise as described in detail in the introduction of this paper, rendering the automated registration a hard task.

In most of the previous works, the methods relied on morphology-based feature extraction to work while the datasets used were homogeneous. The proposed method presents a fast and robust framework to aid clinicians to compare pullbacks acquired at different time points more efficiently. The presented results on the synthetic data demonstrate much better alignment performance than the state-of-the-art, while the experiments on in-vivo data resulted in a worse longitudinal registration performance (see Table 1) but even or better axial registration performance than the state-of-the-art (see Table 2). This performance drop between the synthetic and in-vivo experiments could be attributed to the heterogeneity of the data, especially since our data came from two clinical sites. Notably, other studies do not report such heterogeneity in their datasets. Additionally, there was an increased image similarity after applying the two-stage registration process regardless the time interval between the baseline and follow-up examinations.

The proposed method allows the utilisation, analysis and integration of arterial imaging data facilitating the accurate evaluation of the arterial changes, the disease progress and the treatment approach. This method has been integrated in the 3D reconstruction and plaque characterization tool, a dedicated software tool used for the creation of 3D arterial models. The tool is part of the InSilc platform in silico pipeline, employing novel multi-disciplinary and multi-scale prediction models of stent performance in the acute, short and medium/long term, serving the in silico clinical trials vision and future application.

However, a limitation of this study regards the data and its ground truth. Due to the nature of the data, it is hard, even for a well-trained clinician, to provide accurate one-to-one frame correspondence between pullbacks. Similar to the other studies [2,3,6,7,8,10], the longitudinal ground truth of our dataset does not infer a one-to-one correspondence between all frames of each pair of pullbacks, but regards some frames with annotated landmarks, such as bifurcations and calcification areas.

Another limitation is that the longitudinal registration step utilizes a cost function that is based on the pixel intensities and does not take into consideration structural information of the vessel. This was based on our design principle to develop a fast registration method that does not depend on any computational demanding morphology-based feature extraction mechanism, but comes with the disadvantage of not being able to match longitudinal performance when compared to the state-of-the-art (see Table 1), which may be due to the use of image-based measurements. However, in order to examine if indeed the worse performance is caused by the exclusion of morphological features and not due to the heterogeneity of the data, we plan to extend this method in future work by incorporating structural information into the computation of the DTW cost function, such as the lumen’s or adventitia’s contour analysis in order to investigate if the accuracy and robustness of the method can be significantly increased.

## 6. Conclusions

In this study, a method for registering two IVUS pullbacks both longitudinally and axially has been proposed. It was tested on a heterogeneous dataset of 21 pairs of pullbacks from 21 patients, of which 10 consisted of pullbacks corresponding to pre- and post-stent deployment (immediately after the intervention) IVUS examinations and 11 consisted of pullbacks corresponding to pre-stent and follow-up (after 12 months of the stent intervention) IVUS examinations. The proposed method achieved a mean alignment error 4.3 (0.1853) frames per pullback, a rotational error 12.4° (0.93°) and a distance error 0.56 mm (0.0161 mm) regarding the in-vivo (and synthetic) data. In addition, qualitative results have been presented to understand the results of each registration stage of the method.

## Figures and Tables

**Table 1:** Alignment error of longitudinal registration compared to other studies.

Reference	Error
Ours	4.3±3.9
[2]	1.43±0.68
[3]	1.53±0.92
[7]	4.51
[10]	0.57±1.52

**Table 2:** Distance and rotational error of axial registration compared to other studies.

Reference	ΔL (mm)	$\Delta \Phi^{\circ }$
Ours	0.56±0.323	12.4±10.5
[6]	0.75±1.22	9.27±13.52
[7]	-	16.2

**Table 3:** Performance results on synthetic data.

	Alignment Error		Rotational Error	Translational Error
**Reference**	**Distortions 1–3**	**Distortions 1–4**	**Distortions 1–4**	
Ours	0.0942±0.3882	0.1853±0.1644	0.93±2.3	0.67±1.53(pixels) 0.0161±0.0367 (mm)
[2]	0.93±0.44	-	-	-
[3]	0.76±0.29	-	-	-

**Table 4:** Alignment error of longitudinal registration on in-vivo data. The normalized alignment error is computed with reference to the number of registered frames.

Patient	# of Frames Pullback 1	# of Frames Pullback 2	# of Registered Frames	# of Landmarks	Error (Frames)	Normalized Error
1	155	118	118	5	7.4±0.5	0.063
2	27	36	30	3	3±0.8	0.1
3	84	49	59	5	9.8±3.5	0.166
4	133	113	115	6	1±0	0.009
5	100	82	84	6	3±2.8	0.036
6	77	64	77	5	2.4±0.8	0.031
7	149	152	151	5	2.6±0.5	0.017
8	142	154	140	6	3.7±1.1	0.026
9	83	95	91	9	2.88±2.7	0.032
10	107	93	98	7	1.6±0.7	0.016
11	117	122	117	5	2.2±0.4	0.019
12	98	104	100	6	1±0.8	0.01
13	169	186	169	7	7.9±2.5	0.047
14	115	111	116	5	1.6±0.7	0.014
15	173	144	146	7	8.5±1.8	0.058
16	161	170	139	6	0±0	0
17	212	127	127	5	10±0	0.079
18	143	133	137	7	11.3±2.7	0.082
19	95	121	95	0	-	-
20	67	124	84	5	1.2±1.5	0.014
21	140	108	76	5	4±2	0.053

**Table 5:** Distance and rotational error of axial registration on in-vivo data.

Patient	ΔL (mm)	$\Delta \Phi^{\circ }$
1	0.437±0.136	12.1±3.2
2	0.408±0.12	7.16±3.76
3	0.478±0.11	7.55±4.05
4	0.96±0.15	33.7±12.14
5	0.6±0.109	10.36±2.74
6	0.414±0.0108	13.2±0.5
7	0.588±0.041	24.5±1.71
8	0.69±0.358	28.78±14.9
9	0.626±0.325	15.8±12.4
10	0.308±0.22	3.13±3.8
11	0.618±0.48	16.7±14.2
12	0.348±0.184	7.61±6.31
13	0.256±0.091	4.46±2.26
14	0.839±0.135	14.426±5.31
15	0.495±0.34	12±11.6
16	0.389±0.121	5.6±3.1
17	0.486±0.141	6.9±4.9
18	0.999±0.313	20.12±6.6
19	No landmarks	
20	0.368±0.232	9.44±4.9
21	0.5658±0.207	9.22±5.28

**Table 6:** Dataset comparison with other studies.

Ref	Dataset	Task	Ground Truth	Synthetic Data	Morphological Feature Extraction	Stent
Ours	21 IVUS-IVUS	Longitudinal and Axial Registration	Yes	Yes	No	Yes
[2]	13 IVUS-IVUS	Longitudinal Registration	Yes	Yes	Yes	Yes
[3]	21 IVUS-IVUS	Longitudinal Registration	Yes	Yes	Yes	Yes
[4]	14 IVUS-Histology	Axial Registration	Yes	No	Yes	No
[6]	29 VH IVUS-VH IVUS	Longitudinal and Axial Registration	Yes	No	Yes	No
[7]	12 VH IVUS-OCT	Longitudinal and Axial Registration	Yes	No	Yes	No
[8]	31 IVUS-IVUS	Longitudinal and Axial Registration	No	No	Yes	No
[10]	28 IVUS-IVUS	Longitudinal and Axial Registration	Yes	No	Yes	Yes

## Data Availability

The data presented in this study are available on request from the corresponding author.

## Abbreviations

The following abbreviations are used in this manuscript:

IVUS	Intravascular Ultrasound
DTW	Dynamic Time Warping
2D	Two-Dimensional
3D	Three-Dimensional
ECG	Electrocardiogram
CDF	Cumulative Distribution Function
CC	Cross Correlation
HS	Harmony Search
MI	Mutual Information
